# Migration of a foreign object to the parapharyngeal space: an unusual factitious disorder^☆^

**DOI:** 10.1016/j.bjorl.2016.05.004

**Published:** 2016-06-15

**Authors:** Senol Comoglu, Necati Enver, Comert Sen, Levent Aydemir

**Affiliations:** Istanbul University, Istanbul Faculty of Medicine, Department of Otorhinolaryngology and Head & Neck Surgery, Istanbul, Turkey

## Introduction

Foreign body removal is a common procedure in Otorhinolaryngological (ORL) practice.^1^ It is common in pediatric age, however, it may also be seen in adults. In adults fish bones and swallowed Foreign Bodies (FB) are mostly seen. The most common places where FB are found are the palatine tonsils, vallecula and piriform sinuses; however, FB can, although rarely, migrate to deep neck spaces, like the parapharyngeal space.^2^ This case presents migration of a factitious foreign body to the Parapharyngeal Space (PPS).

## Case report

**Figure 1:**
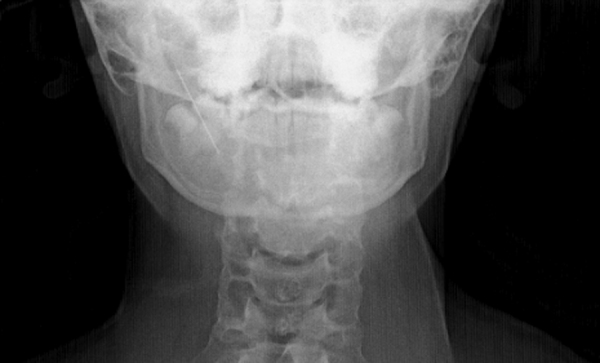
Anteroposterior cervical X-ray.

**Figure 2:**
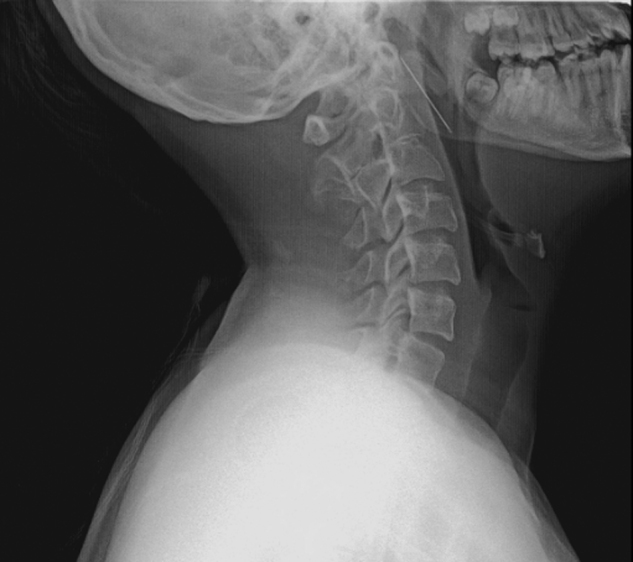
Lateral cervical X-ray.

A 14 year-old girl presented to our clinic with odynophagia and neck pain. The patient's medical history included several foreign body removals from the head and neck region, such as the external ear canal and nostril. Three days prior attending to our clinic the patient had tried to locate pebbles into her lacrimal canal. After further questioning she admitted that she had plunged a sewing needle into the right buccal area in her mouth 1 week ago. Physical examination showed no signs of penetration. Lateral and antero-posterior neck X-ray revealed one needle-like FB in the right parapharyngeal space (Figure 1, Figure 2). On contrast-enhanced Computed Tomography (CT) study a FB in the right-hand side parapharyngeal space was observed. It was lying in the level of C1–C3 vertebra, approximately 4.4 mm medial to the external carotid artery, between the internal and external carotid arteries, with the sharp side superiolateral with a 30° angle.

**Figure 3:**
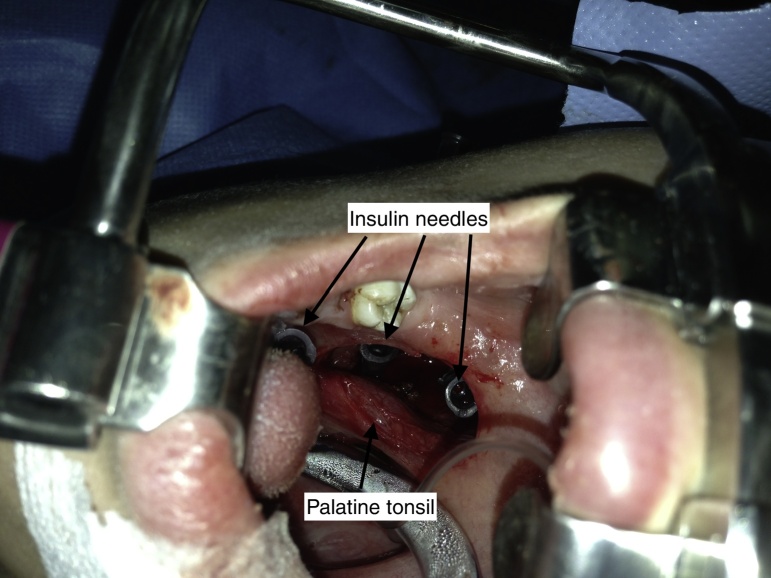
Intraoperative view: insulin syringe needles used as landmarks in intraoperative X-ray imaging and tissue dissection.

**Figure 4:**
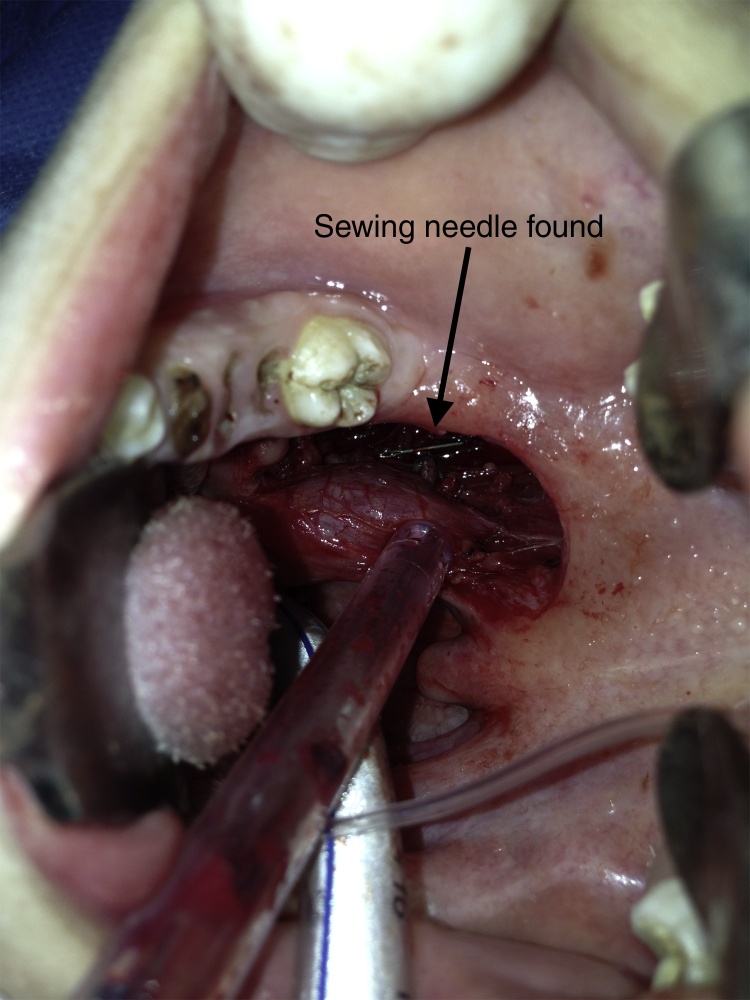
Intraoperative view: sewing needle found in the parapharyngeal space.

**Figure 5:**
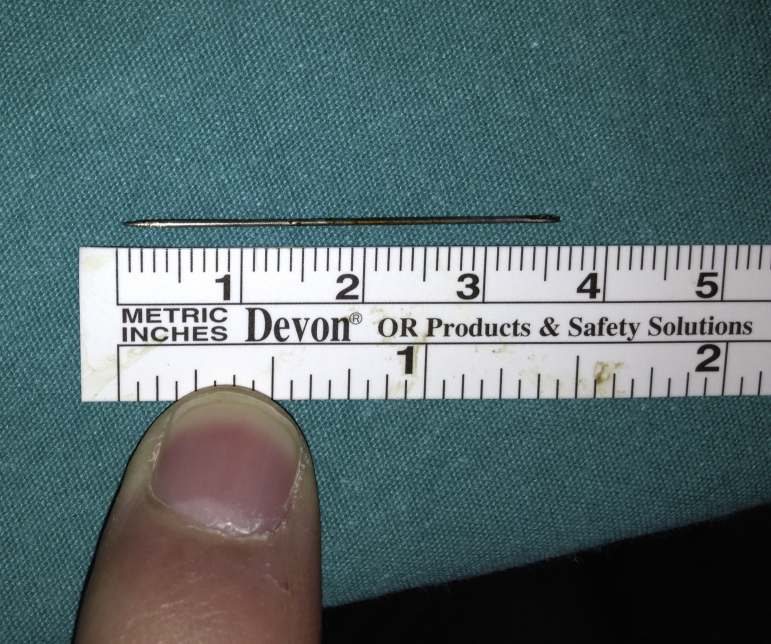
Extracted sewing needle (3.6 cm long).

Because of the proximity to main vascular structures and the near localization to the palatine tonsillar bed a transoral approach was decided to extract the FB. Starting the operation with a 4 cm long vertical incision lateral to the right anterior tonsillar pillar, the parapharyngeal space was reached. The FB could not be found by dissecting the tissue. Therefore we decided to position insulin syringe needles into the dissection area to create artificial radiopaque landmarks and perform intraoperative X-rays to the neck from several angles. The localization of the FB was predicted with the assistance of these images and dissection was performed using the four insulin syringe needles as landmarks (Fig. 3). The needle was found and removed without any complication (Figure 4, Figure 5).

After surgery prophylactic antibiotherapy was given for one week and oral intake was allowed at postoperative Day 1. Discharging the patient at postoperative Day 4 the patient was referred to the Department of Child and Adolescent Psychiatry and diagnosed as having a factitious disorder during follow-up.

## Discussion

Most common pharyngeal FBs in adult patients are fish bones. They are mostly swallowed accidently and can be easily removed under local anesthesia. Other non-organic foreign bodies in deep neck spaces have different etiologies. Penetrant neck traumas, traffic accidents, ingested foreign bodies, intraoral local anesthesia complications and factitious disorders are some of them. In some cases migration of FB to deep neck spaces can occur. The migration is triggered by contraction of neck muscles and viscera and does not always follow anatomical planes.^2^ Foreign bodies in the neck spaces sometimes can even cause retropharyngeal abscess, thyroid abscess and neck lumps. More catastrophic complications are carotid artery and jugular vein lacerations.^3^

The face and ear are the two most common affected sides in ORL practice for factitious symptoms.^4^ Foreign bodies in the external ear canals and nostrils and even facial emphysema is one of probable findings, but a FB in the soft tissue of the neck is very rare. To date there has been no case of a needle migration to the parapharyngeal space as a result of factitious behavior in the literature.^5^ The PPS occupies the space between the muscles of mastication and the muscles of deglutition. It is shaped like a pyramid, inverted with its base at the skull base, with its apex inferiorly pointing to the greater cornu of the hyoid bone. The PPS is limited anteriorly by the pterygomandibular raphe and pterygoid fascia and posteriorly by the cervical vertebrae and prevertebral muscles. The medial border of the PPS is the pharynx, and the lateral border comprises the ramus of the mandible, the medial pterygoid muscle, and the deep lobe of the parotid gland.^2^

Parapharyngeal space lesions can be explored via transcervical, transparotid, transmandibular and transoral approaches. The most effective and suitable method can be chosen according the location of the FB. In our case, the needle was located between the external carotid artery and the pharyngeal wall, which led us to choose a transoral approach.

Radiography is not always necessary for FBs in the pharynx. FBs lodged in the pharynx can be easily seen with direct laryngoscopy or eusophagoscopy. However, migrating-perforating FBs need to be visualized to confirm their presence. Plain anterio-posterior and lateral neck radiography is an inexpensive and effective tool for diagnosis of radiopaque FBs.^6^ CT scan is commonly necessary for showing the relation with important anatomic structures and to clarify the exact location of the FB. 3D reconstruction of CT is very helpful for preoperative planning of surgery.^7^ As it was used in our case, fluoroscopy is an important surgical aid during surgery for radiopaque FBs. Ultrasonography is another imaging option, which has successfully been used intraoperatively to localize FBin the neck.^1^ With a transcervical approach, one has a chance to make a wide incision for exposition. Although it achieves better esthetic results, transoral surgery has disadvantages like limited area for surgical intervention.

FBs in the neck require surgical exploration due to the risk of infection and perforation of the anatomic structures like the internal carotid artery and internal jugular vein. However, there are no certain criteria for the timing of surgery; the general trend is to undertake surgery in a short period of time after symptoms start. No correlation has been shown between mortality and duration of retention of foreign bodies.^8^ Zhao et al. presented a delayed case of glass fragment that punctured the internal jugular vein and cranial nerves which caused neurologic deficits. In their case they surgically removed an FB 3 weeks after the accident and found fibrous tissue around the FB. They emphasized that in some cases FBs can be followed up with serial CT scans and delayed surgery can be performed.^9^ Bilish et al. showed that after surgical failures for foreign body removal from the soft tissue of the neck, serial CT scans can be useful for understanding migration patterns and the final location of foreign bodies for subsequent surgeries.^10^

## Conclusion

This case demonstrates that physicians should keep in mind that foreign bodies in the pharynx could result from a factitious disorder and can migrate into the parapharyngeal space. We report our experience with the intraoperative management of a FB in the PPS. While a preoperative CT scan is useful, especially for radiopaque FBs, intraoperative imaging should also be considered. Having in mind the challenging task of approaching the PPS via transoral approach, one should have in mind creating artificial landmarks for guidance significantly facilitates the surgery. There are no criteria defining surgical timing for foreign bodies in the PPS.

## Conflicts of interest

The authors declare no conflicts of interest.
